# Comparative Estimates of Crude and Effective Coverage of Measles Immunization in Low-Resource Settings: Findings from Salud Mesoamérica 2015

**DOI:** 10.1371/journal.pone.0130697

**Published:** 2015-07-02

**Authors:** K. Ellicott Colson, Paola Zúñiga-Brenes, Diego Ríos-Zertuche, Carlos J. Conde-Glez, Marielle C. Gagnier, Erin Palmisano, Dharani Ranganathan, Gulnoza Usmanova, Benito Salvatierra, Austreberta Nazar, Ignez Tristao, Emmanuelle Sanchez Monin, Brent W. Anderson, Annie Haakenstad, Tasha Murphy, Stephen Lim, Bernardo Hernandez, Rafael Lozano, Emma Iriarte, Ali H. Mokdad

**Affiliations:** 1 Division of Epidemiology, University of California, Berkeley, California, United States of America; 2 Institute for Health Metrics and Evaluation, University of Washington, Seattle, Washington, United States of America; 3 Salud Mesoamérica 2015/Inter-American Development Bank, Panama City, Panama; 4 Instituto Nacional de Salud Pública, Cuernavaca, Mexico; 5 George Washington University, Washington, District of Columbia, United States of America; 6 El Colegio de la Frontera Sur-Mexico, San Cristóbal de Las Casas, Chiapas, Mexico; 7 County of Napa Health and Human Services, Department of Public Health, Napa, California, United States of America; 8 School of Social Work, University of Washington, Seattle, Washington, United States of America; Melbourne School of Population Health, AUSTRALIA

## Abstract

Timely and accurate measurement of population protection against measles is critical for decision-making and prevention of outbreaks. However, little is known about how survey-based estimates of immunization (crude coverage) compare to the seroprevalence of antibodies (effective coverage), particularly in low-resource settings. In poor areas of Mexico and Nicaragua, we used household surveys to gather information on measles immunization from child health cards and caregiver recall. We also collected dried blood spots (DBS) from children aged 12 to 23 months to compare crude and effective coverage of measles immunization. We used survey-weighted logistic regression to identify individual, maternal, household, community, and health facility characteristics that predict gaps between crude coverage and effective coverage. We found that crude coverage was significantly higher than effective coverage (83% versus 68% in Mexico; 85% versus 50% in Nicaragua). A large proportion of children (19% in Mexico; 43% in Nicaragua) had health card documentation of measles immunization but lacked antibodies. These discrepancies varied from 0% to 100% across municipalities in each country. In multivariate analyses, card-positive children in Mexico were more likely to lack antibodies if they resided in urban areas or the jurisdiction of De Los Llanos. In contrast, card-positive children in Nicaragua were more likely to lack antibodies if they resided in rural areas or the North Atlantic region, had low weight-for-age, or attended health facilities with a greater number of refrigerators. Findings highlight that reliance on child health cards to measure population protection against measles is unwise. We call for the evaluation of immunization programs using serological methods, especially in poor areas where the cold chain is likely to be compromised. Identification of within-country variation in effective coverage of measles immunization will allow researchers and public health professionals to address challenges in current immunization programs.

## Introduction

Measles is an infectious vaccine-preventable disease that causes more than 125,000 worldwide deaths annually, most in children under 5 years of age [1]. Although endemic measles transmission was first interrupted in Mexico in 1997 and there have been no confirmed cases in Nicaragua since 1995, both countries remain vulnerable to imported cases and outbreaks, particularly in population clusters with low vaccination coverage [2]. Lapses in coverage in poor, rural areas of Mexico led to a devastating epidemic in 1989 and to reintroduction of endemic transmission in April 2000 [2]. Accurate measurement of immunization coverage is critical to preventing future outbreaks; however, concerns have been raised about the accuracy of current metrics [3–5]. In both countries, existing data on vaccination coverage comes from national health surveys [6–11], which typically capture data from child health cards and rely on caregiver recall when cards are unavailable. Differences between survey-based coverage estimates and validating sources such as medical records indicate a large degree of inaccuracy, ranging from -40 to +56 percentage points [12]. Administrative estimates are subject to error and bias from both numerator data (number of doses distributed) and denominator data (the number of persons who should have received the vaccine). Most importantly, these sources do not capture the gap between crude (vaccination) and effective coverage (seroconversion) [13].

There is growing interest in the use of seroepidemiology to monitor the effective coverage of immunizations [14–17]. Dried blood spots (DBS), drops of capillary blood dried on filter paper, are an affordable, minimally invasive method of obtaining blood in non-clinical settings and are being used to measure biomarkers such as antibodies to infectious agents [18,19]. Three previous studies [20–22] have validated methods for analyzing DBS for the presence of measles-specific immunoglobulin G (IgG). These methods cannot distinguish between naturally occurring and vaccine-induced antibodies, but in Mexico and Nicaragua where measles incidence is low [23,24], the vast majority of positive cases can be attributed to immunization.

To our knowledge, no previous studies of measles serology in Nicaragua exist at either the national or subnational level. In Mexico, several national health surveys [8,10] have collected DBS or blood samples and one [25] recently quantified the prevalence of measles antibodies for children aged 1 to 4 years old. This study reported a national seroprevalence of 98.3%. However, there is substantial evidence that national averages in health service delivery in Mexico mask significant subnational variation [26–28]. Very little is known about the accuracy of crude immunization coverage estimates or the effectiveness of immunization programs, particularly in the poorest areas. Moreover, little is known about why disparities between crude and effective coverage may exist, and in which populations these gaps may be concentrated. This evidence gap is particularly worrisome given that the communities most likely to experience outbreaks are also the populations for whom the least is known about population protection against measles.

To address this dearth of subnational serological evidence, we quantified the difference between crude coverage and effective coverage of measles immunization among children aged 12 to 23 months in poor areas of Mexico and Nicaragua and identified individual, maternal, household, community, and health facility characteristics that predict gaps between crude coverage and effective coverage.

## Materials and Methods

### Study design and participants

This study is part of the baseline evaluation of Salud Mesoamérica 2015, a results-based financing initiative to improve child and maternal health in the poorest populations in eight Mesoamerican countries. Additional information on Salud Mesoamérica 2015 methodology is available elsewhere [29].

In designated municipalities representing the poorest wealth quintile in Nicaragua and the Mexican state of Chiapas, we sampled localities (segments) with probability proportional to size. In selected segments we conducted a complete housing and population census. We used the census to identify households with women aged 15 to 49 years and/or children under five, and randomly sampled 30 such households per segment for interviews related to maternal and child health. We captured information related to household characteristics, access to health facilities, exposure to social programs and health interventions, current health status, recent illnesses, reproduction, contraception, antenatal, delivery, and postnatal care, child nutrition, and immunization. Caregivers of all children were asked to recall, if possible, the type and number of immunizations each child received. Surveyors then asked to see each child’s health card, if one was available, and recorded the names and dates of administered immunizations. Professional nurses received a rigorous, standardized training on anthropometric measurement and collection blood samples for biomarkers. Once trained, they obtained written informed consent from caregivers to measure the height, weight, and hemoglobin level of children under 5 years and to collect DBS samples from all children aged 12 to 23 months (nominally one year old). DBS were obtained specifically for one-year-olds, because measles vaccine is prescribed at 12 months, so all children in this age group should be immunized but not yet experience waning immunity [30]. DBS samples were obtained from 71.9% (n = 1,134) and 86.2% (n = 454) of children aged 12 to 23 months in Mexico and Nicaragua, respectively.

Procedures for the collection and analysis of DBS are described in detail elsewhere [22]. Briefly, drops of blood from a heel or finger stick were collected on demarked circles on filter paper. Samples were dried at room temperature and then inserted into a plastic bag with a desiccant, and shipped refrigerated to the National Institute of Public Health Laboratory in Cuernavaca, Mexico, where they were stored at -20°C until processing. DBS were re-liquefied using phosphate buffered saline and assayed for the presence of measles-specific immunoglobulin G antibodies using the HUMAN Worldwide Diagnostics Measles IgG ELISA kit (Wiesbaden, Germany). Because performance of the assay was proven for use with venous whole blood but not re-liquefied DBS, we conducted a study to evaluate the accuracy, precision, and reliability of the ELISA procedure with DBS and found the test was 100% sensitive and 96.8% specific [22]. DBS samples in the current study were analyzed in the same laboratory with the same procedures, reagents, and technicians as the validation study. In accordance with kit instructions, each assay was run with two positive and two negative controls to confirm assay acceptability and to establish plate-specific positive and negative cutoff values. Samples with assay results falling between the positive and negative cutoff values (149 samples) were re-assayed and the second result was taken as the final result. Forty-three samples remained indeterminate and were excluded from the analysis.

We also conducted interviews in a random sample of 180 health centers and captured information on facility policies, staffing, infrastructure, vaccination procedures and supplies, and functionality of equipment used to store vaccines. We measured the temperature of all refrigerators at the time of the survey, obtained either from thermometer readings that could be viewed outside the refrigerator or from temperature monitoring charts when the thermometer was located inside the refrigerator.

Due to safety concerns in the Nicaraguan states of Jinotega and the North Atlantic Region, data collection was halted before all selected localities and health facilities could be surveyed. Therefore, surveys were completed with 2,052 households (83% of targeted households) and 64 health facilities (71% of targeted facilities). To confirm that no bias was introduced, we compared socioeconomic, health services, and demographic characteristics of surveyed and non-surveyed areas using the most recent national census and found no differences between them.

Permission to implement this research project was obtained from the Health Institute of the State of Chiapas and the Nicaragua Ministry of Health. Ethical approval for this study was obtained from the institutional review boards of the University of Washington, the Mexico National Institute of Public Health, El Colegio de la Frontera Sur-Mexico, and the National Autonomous University of Nicaragua. We obtained written informed consent from health facility managers for facility surveys and from household survey participants. We also obtained written informed consent from the mother or guardian of each child providing dried blood spot samples. All ethical and institutional review boards approved the consent procedures. The study was done in compliance with national regulatory and ethics guidelines.

### Outcomes

We defined crude coverage of measles immunization as the proportion of children with at least one documented measles immunization on their health card, and recall-based coverage as the proportion of children whose caregivers self-reported that the child had received at least one measles immunization. We defined survey-based coverage as the proportion of children meeting the criteria for crude (card-based) coverage, recall-based coverage, or both. We defined crude coverage of complete immunization as the proportion of children with immunization records for all nationally recommended vaccines for children 12 to 23 months old. Finally, we defined effective coverage of measles immunization as the proportion of children with a positive DBS assay for measles-specific IgG antibodies.

### Statistical analyses

First, we examined concordance between three sources of vaccination information: caregiver recall, health cards, and DBS. Next, we calculated the kappa, sensitivity, specificity, and positive and negative predictive values of health card documentation in predicting serostatus.

Of particular interest are children with health card documentation of measles immunization who lack antibodies. To identify potential drivers of this discrepancy, we restricted our analysis to health card-positive children (N = 676 in Mexico and N = 262 in Nicaragua) and used logistic regression to identify characteristics that increase the likelihood that a card-positive child will lack antibodies. We tested a wide variety of individual, maternal, household, community and health facility factors, and report crude and adjusted odds ratios. Because the number of children studied in regression analyses was relatively small, we lacked the statistical power to examine all measured, potentially relevant factors at the same time. Hence, we tested several combinations of variable sets. Variable selection for the final models was based on consistency in the exposure-outcome relationship across models and selection of factors that were most likely to explain differences in serostatus among card-positive children according to previously established associations and vaccination and local area experts.

To coordinate household and health facility data, we linked the two surveys using the caregiver self-reported name of the health facility attended for vaccination or the average characteristics of facilities serving the region. Because the SM2015 target populations cover a large geographic region and because SM2015 interventions involve primarily Ministry of Health (MOH)-funded facilities, we surveyed a representative sample of 90 of 826 and 64 of 240 MOH facilities serving the study populations in Mexico and Nicaragua, respectively. For this reason, not all households could be linked to surveyed health facilities by name. When the name was not reported or the named facility was not interviewed, we assigned the average characteristics of surveyed health facilities that offered vaccination and served the area of residence of the child (municipalities in Mexico and Local Integrated Healthcare System geographic regions in Nicaragua). We matched 25% and 30% of card-positive children by name in Mexico and Nicaragua, respectively, and the remaining children by region.

In all analyses, we excluded 46 children in Mexico and 18 children in Nicaragua whose health cards indicated that DBS collection occurred too soon (within 28 days) after vaccination for development of a detectable IgG antibody titer [30]. We retained 61 children in Mexico and 20 children in Nicaragua with DBS samples whose health cards indicated previous measles vaccination, but no date was recorded. While it is possible that these children received measles immunization within 28 days of interview, the vast majority (94%) of children with dates on their health cards were sampled more than 28 days after vaccination, and therefore it is unlikely that the DBS samples for children without dates were taken too soon after vaccination.

We used Stata (version 13.1) and R (version 3.0.2). All coverage estimates and regression analyses account for the complex survey design using survey settings in Stata and the survey package in R.

To confirm our findings, we conducted several sensitivity analyses. First, we tested the variability of our results when excluding children vaccinated between seven and 35 days before DBS collection. Second, we tested regression models among both card-positive and recall-positive children rather than card-positive children only. Third, we tested regression models restricted to the subsample of children matching to health facilities by name.

## Results

In Mexico, between July 25, 2012 and May 18, 2013, we surveyed 5,428 households with 6,988 women of reproductive age and 1351 children aged 12 to 23 months and collected DBS from 1,134 children. In Nicaragua, between March 1, 2013 and September 3, 2013, we surveyed 2,052 households with 1,713 women of reproductive and 475children aged 12 to 23 months and collected DBS from 454 children aged 12 to 23 months old. A majority of mothers were married or partnered homemakers in their 20s with primary education and an average of 3.3 and 2.6 children in Mexico and Nicaragua, respectively (Table 1).

**Table 1:** Household characteristics of the DBS study sample.

Univariate characteristic			Mexico	Nicaragua
			% or mean (95% confidence interval)	% or mean (95% confidence interval)
**Child**	Age <18 months		47.3 (44.2–50.4)	34.0 (28.0–40.6)
	Female		51.0 (47.6–54.3)	51.3 (44.8–57.9)
	Child health:	Excellent	11.4 (9.1–14.2)	16.9 (12.1–22.9)
		Very good	15.2 (12.9–17.8)	24.3 (19.7–29.6)
		Good	51.8 (48.4–55.1)	29.6 (24.3–35.5)
		Moderate	19.3 (16.7–22.1)	26.2 (20.7–32.4)
		Poor	2.3 (1.4–3.7)	3.1 (1.5–6.0)
	Diarrhea in last 2 weeks		17.6 (15.4–20.0)	18.8 (14.0–24.9)
	Fever in last 2 weeks		21.7 (18.8–24.9)	18.1 (13.9–23.3)
	Anemic		33.5 (29.3–38.1)	46.7 (40.9–52.5)
	Underweight^1^		8.7 (6.7–11.2)	1.9 (0.6–5.6)
	Wasted^2^		2.0 (1.3–3.3)	0.2 (0.0–1.7)
**Maternal**	Age (years):	15–19	11.5 (9.3–14.1)	14.4 (10.6–19.3)
		20–24	28.3 (25.5–31.2)	31.4 (26.3–36.9)
		25–29	27.3 (24.3–30.6)	24.7 (20.4–29.5)
		30–34	17.5 (15.2–20.0)	14.3 (9.9–20.1)
		35–39	10.4 (8.6–12.6)	10.2 (7.1–14.5)
		40–44	4.2 (3.0–5.8)	4.4 (2.5–7.7)
		45–49	0.8 (0.4–1.6)	0.6 (0.1–2.3)
	Age at first birth <20 years		65.9 (62.3–69.3)	67.6 (61.7–73.1)
	Parity (mean)		3.3 (3.1–3.5)	2.6 (2.3–2.8)
	Marital status:	married	35.1 (30.7–39.7)	31.2 (25.5–37.6)
		single	1.7 (1.0–2.8)	18.0 (13.0–24.5)
		open union	58.0 (53.3–62.5)	45.3 (39.9–50.8)
		others	5.3 (4.0–6.9)	5.4 (3.3–8.7)
	Literate		56.9 (52.5–61.3)	71.8 (65.7–77.2)
	Education:	none	15.2 (12.6–18.3)	11.4 (7.7–16.6)
		literacy course	2.8 (1.6–4.8)	48.8 (41.7–55.9)
		primary	51.9 (47.8–56.0)	28.2 (23.4–33.5)
		secondary +	30.1 (26.3–34.2)	11.6 (6.8–19.2)
	Occupation:	paid employee	5.5 (3.9–7.7)	12.5 (8.6–17.9)
		homemaker	93.3 (91.0–95.1)	84.5 (78.9–88.8)
		other	1.2 (0.6–2.2)	3.0 (1.3–6.5)
	Exposure to mass media		70.4 (65.7–74.7)	88.5 (83.0–92.4)
	Met with Promotora in last month		19.1 (15.5–23.4)	1.4 (0.6–3.5)
	In good health		61.0 (56.4–65.4)	64.7 (59.2–69.8)
	Speaks indigenous language		66.1 (58.5–73.0)	12.1 (5.5–24.4)
**Household**	Father lives in home		92.1 (90.3–93.7)	72.9 (65.9–78.9)
	Household size (mean)		5.9 (5.7–6.2)	5.9 (5.5–6.2)
	Monthly household expenditure (pesos):	0–499	10.5 (8.2–13.4)	
		500–999	18.3 (15.4–21.7)	
		1000–1999	33.0 (29.5–36.7)	
		2000–4999	32.9 (29.0–36.9)	
		5000 +	5.3 (3.8–7.2)	
	Monthly household expenditure (córdobas):	0–499		2.8 (1.2–6.7)
		500–999		5.7 (3.5–9.0)
		1000–1999		22.0 (16.7–28.4)
		2000–3499		26.4 (21.9–31.4)
		3500–5499		20.3 (15.0–26.9)
		5500–8999		14.1 (10.1–19.3)
		9000+		8.7 (5.5–13.5)
	Asset score (mean)		0.2 (0.2–0.2)	24.9 (23.4–26.3)
	Interview in Spanish		78.8 (72.8–83.8)	97.9 (93.8–99.3)
	Indigenous language spoken in household		69.4 (61.9–75.9)	13.2 (6.1–26.2)
	Travel time (minutes) to health facility:	< 15	31.7 (26.4–37.5)	30.8 (22.8–40.2)
		15–29	23.3 (19.9–27.0)	21.7 (16.7–27.6)
		30–44	23.6 (19.8–27.8)	18.3 (13.5–24.3)
		45–59	2.2 (1.3–3.6)	1.4 (0.4–4.9)
		60 +	19.3 (14.1–25.9)	27.8 (20.5–36.4)
	Enrolled in Seguro Popular health insurance		86.2 (83.5–88.5)	
	Enrolled in Oportunidades program		64.7 (59.7–69.4)	
	Improved water source		87.6 (81.8–91.8)	82.6 (77.2–86.9)
	Improved sanitation		64.1 (56.9–70.7)	14.7 (9.7–21.6)
**Community**	Urban		34.5 (27.1–42.8)	34.9 (22.5–49.8)
	Altitude:	<500m	16.6 (11.1–24.1)	37.0 (24.1–52.0)
		500–999m	25.9 (19.0–34.2)	46.7 (33.3–60.5)
		1000m +	57.6 (48.6–66.0)	16.4 (9.2–27.5)
			N = 1134	N = 454

Estimates are survey weighted.

^1^ Underweight: proportion of children with weight for age below minus two standard deviations from median of World Health Organization reference population.

^2^ Wasted: proportion of children with weight for height below minus two standard deviations from median of World Health Organization reference population. m: meters.

A majority of health facilities were ambulatory clinics (Table 2). A substantial proportion lacked basic infrastructure such as running water and electricity. While most facilities offer vaccination, only 49% in Mexico and only 84% in Nicaragua routinely store vaccines. Some facilities reported challenges in vaccine supply. For example, 49% of facilities in Mexico and 24% of facilities in Nicaragua reported not always receiving the number of vaccines ordered and 21% in Mexico and 8% in Nicaragua did not have measles-mumps-rubella vaccine in stock on the day of the survey. Challenges in cold chain management were also present: 16% of facilities in Mexico and 44% of facilities in Nicaragua had at least one broken refrigerator or cold box and 20% (Mexico) and 7% (Nicaragua) had at least one vaccine storage unit outside of the appropriate temperature range (2–8°C) on the day of the survey.

**Table 2:** Health facility characteristics of the DBS study sample.

Univariate characteristic		Mexico		Nicaragua	
		% or mean (95% confidence interval)	N	% or mean (95% confidence interval)	N
**Roles and policies**	Ambulatory facility	66.7 (55.9–76.3)	90	85.9 (75.0–93.4)	64
	Receives referrals	30.0 (20.8–40.6)	90	43.8 (31.4–56.7)	64
	Secretaría de Salud accredited	57.8 (46.9–68.1)	90	92.2 (82.7–97.4)	64
	Offers child services	91.1 (83.2–96.1)	90	100 (94.4–100)	64
	Offers vaccination	88.9 (80.5–94.5)	90	95.3 (86.9–99.0)	64
	Stores vaccines	48.8 (37.4–60.2)	80	78.1 (66.0–87.5)	64
	Routine administrative meetings	80.0 (70.2–87.7)	90	81.2 (69.5–89.9)	64
	Routine medical meetings	58.9 (48.0–69.2)	90	73.4 (60.9–83.7)	64
**Infrastructure**	Running water	76.7 (66.6–84.9)	90	75.0 (62.6–85.0)	64
	Internet access	21.1 (13.2–31.0)	90	10.9 (4.5–21.2)	64
	Electricity	93.3 (86.1–97.5)	90	92.2 (82.7–97.4)	64
	Sufficient electrical power for all equipment	73.8 (63.1–82.8)	84	76.6 (64.3–86.2)	64
	Electricity at all hours	83.1 (72.9–90.7)	84	72.6 (59.8–83.1)	62
	Electricity on all days	65.0 (53.5–75.3)	81	54.2 (40.8–67.3)	59
	Generator	6.7 (2.5–13.9)	84	7.8 (2.6–17.3)	64
	Working, fueled generator	11.9 (5.9–20.8)	84	20.3 (11.0–32.8)	59
	National immunization scheme observed	84.0 (74.1–91.2)	81	68.4 (54.8–80.1)	57
	Vaccination registry observed	87.7 (78.5–93.9)	81	87.9 (76.7–95.0)	58
	1+ broken cold storage observed	15.6 (8.8–24.7)	80	43.9 (30.7–57.6)	57
	1+ broken thermometer observed	23.9 (12.6–38.8)	46	6.5 (0.8–21.4)	31
	Number of refrigerators (mean)	1.2 (0.1–2.3)	38	1.3 (-0.6–3.3)	50
	Number of ice packs observed (mean)	1.7 (0.5–2.8)	77	1.8 (0.7–2.8)	60
	Any refrigerator out of temperature range	20.0 (8.4–36.9)	35	7.0 (1.5–19.1)	43
	Fridge temp recorded 2x/day in last 30 days	77.1 (59.9–89.6)	35	40.4 (27.0–54.9)	52
**Personnel**	Has doctor	82.2 (72.7–89.5)	90	48.4 (35.8–61.3)	64
	# of doctors (mean)	5.0 (-9.5–19.4)	90	2.0 (-8.0–12.0)	64
	# of nurses (mean)	7.1 (-30.9–45.0)	90	5.3 (-22.3–32.9)	64
	# of pediatricians (mean)	0.5 (-3.9–4.9)	90	0.5 (-2.1–3.0)	64
	# of auxiliary nurses (mean)	1.6 (-4.8–8.0)	90	4.2 (-17.8–26.2)	64
	# of social workers (mean)	0.9 (-3.3–5.1)	90	0.1 (-0.8–1.1)	64
	# of lab technicians (mean)	1.1 (-7.5–9.7)	90	0.8 (-4.0–5.6)	64
	# of promotoras (mean)	1.5 (-4.4–7.4)	90	0.4 (-3.5–4.2)	64
	Total # of staff	20.0 (-45.1–85.0)	90	13.4 (-48.1–74.8)	64
**Vaccine supply**	Always receives number of vaccines ordered	51.3 (34.8–67.6)	39	76.0 (61.8–86.9)	50
	Shortage of measles vaccine in last 6 months	5.6 (1.8–12.5)	39	0.0 (0.0–26.5)	50
	Days between vaccine order and receipt	2.5 (-7.8–12.9)	37	1.4 (-1.0–3.8)	50
	During shortage:	43.6 (27.8–60.4)	39	80.0 (66.3–90.0)	50
	makes special order				
	borrows from other facility	28.2 (15.0–44.9)	39	10.0 (3.3–21.8)	50
	does nothing	25.6 (13.0–42.1)	39	6.0 (1.3–16.5)	50
	other	10.3 (2.9–24.2)	39	6.0 (1.3–16.5)	50
	Vaccine orders placed at fixed times (vs as needed)	82.5 (67.2–92.7)	40	69.4 (54.6–81.7)	49
	Anticipated a shortage in last 3 months	52.6 (35.8–69.0)	39	32.0 (19.5–46.7)	50
	MMR vaccine observed in stock	79.2 (65.9–89.2)	53	91.3 (79.2–97.6)	46

Values are percentages unless otherwise noted. 95% confidence intervals are presented in parentheses.

Estimates are not survey weighted. MMR: measles-mumps-rubella.

The health facility survey incorporated skip logic. For each item, N refers to the denominator (i.e., the number of facilities for which the question was relevant).

**Fig 1:**
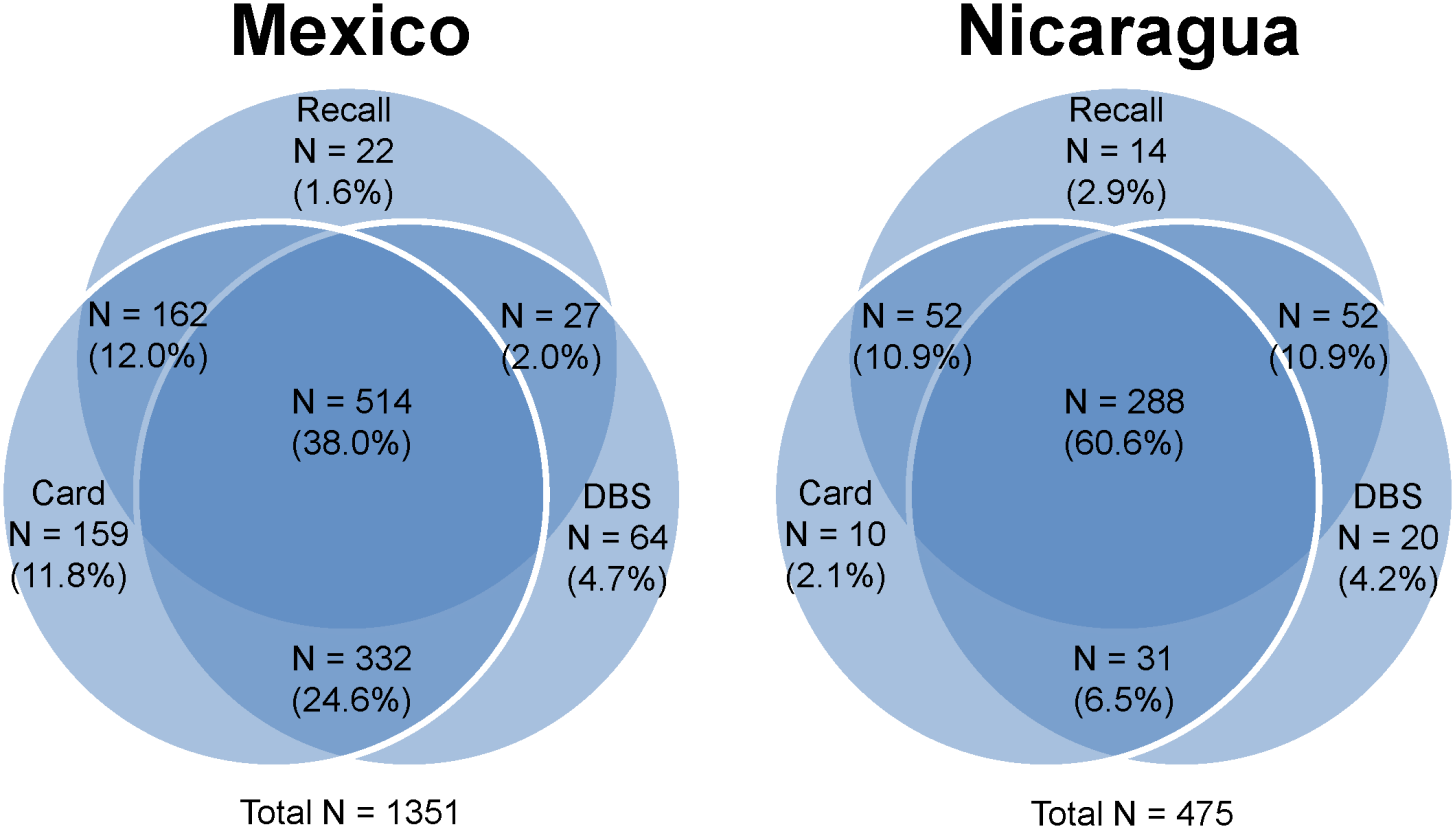
Proportions of children with measles immunization information from caregiver recall, health cards, and dried blood spots. This figure displays the proportion of surveyed children aged 12 to 23 months in Mexico and Nicaragua with measles immunization information from survey and biomarker sources. Many caregivers struggled to recall the type and number of vaccines given, but we were able to collect health card documentation and DBS from a majority of children in both countries. This study focuses primarily on children with both health card and DBS sources, comprising 62.6% of the sample in Mexico and 67.1% of the sample in Nicaragua.

**Fig 2:**
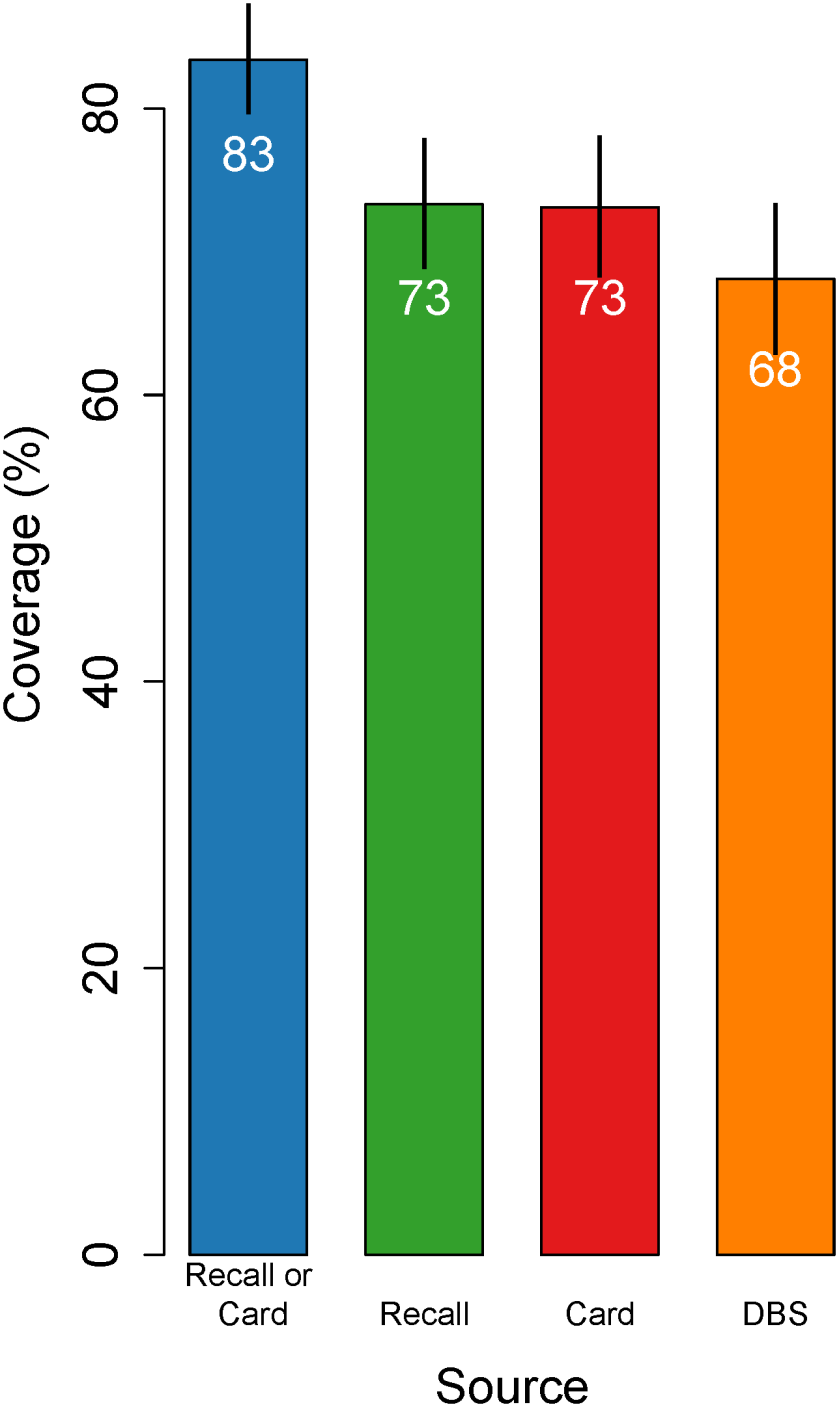
Crude and effective coverage of measles immunization in Mexico. This figure displays estimates of measles immunization coverage based on (1) the combination of child health cards and caregiver recall, (2) caregiver recall only, (3) child health card only, and (4) analyses of dried blood spot samples. Coverage estimates are highest according to the combination of child health card and caregiver recall, and lowest according to dried blood spot samples, and these differences were statistically significant. The figure is restricted to the 552 children with coverage information from all three sources, excluding children with DBS collection within 28 days of vaccination. Lines indicate 95% confidence intervals. Estimates are survey weighted.

**Fig 3:**
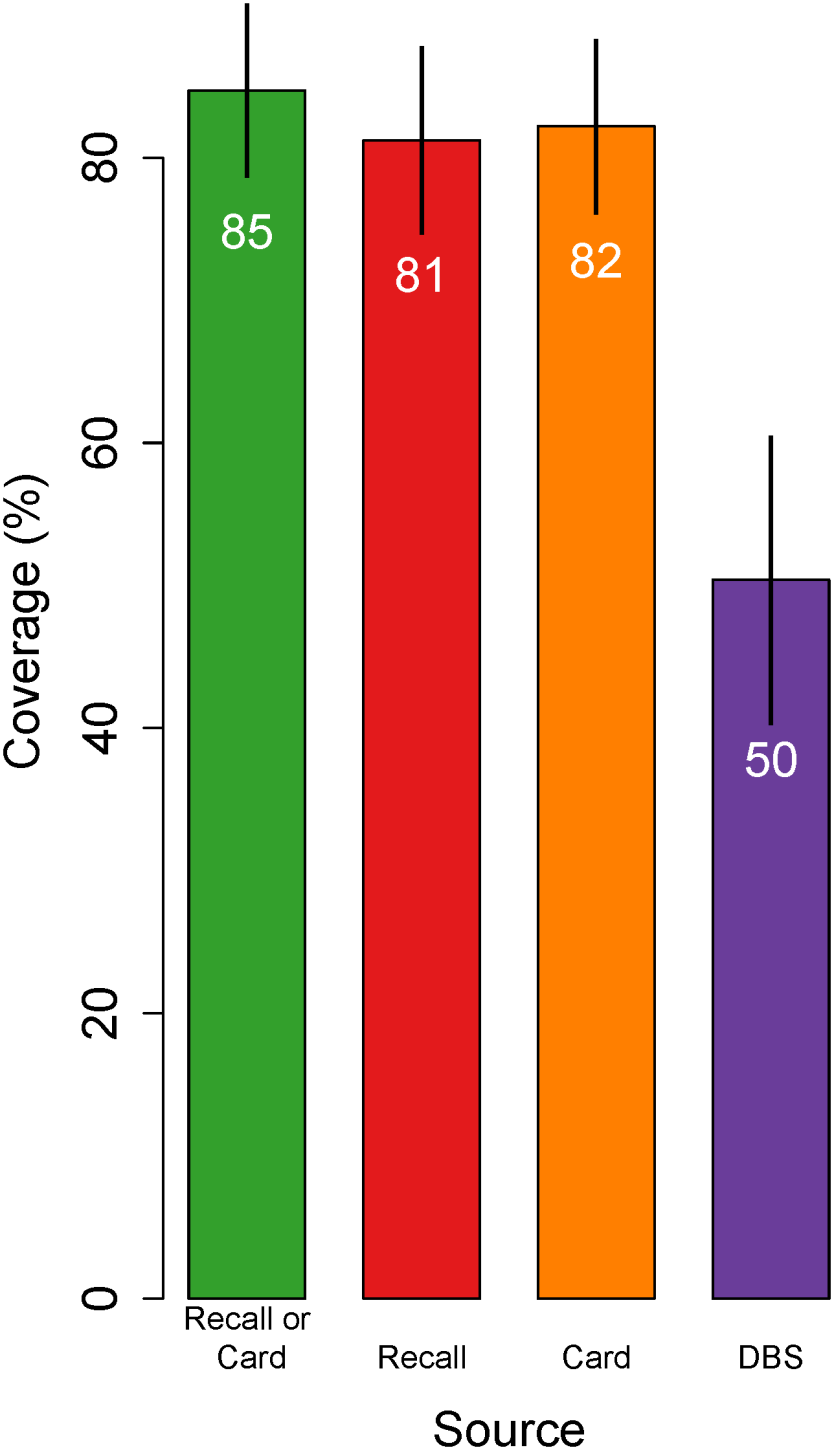
Crude and effective coverage of measles immunization in Nicaragua. This figure displays estimates of measles immunization coverage based on (1) the combination of child health cards and caregiver recall, (2) caregiver recall only, (3) child health card only, and (4) analyses of dried blood spot samples. Coverage estimates are highest according to the combination of child health card and caregiver recall, and lowest according to dried blood spot samples, and these differences were statistically significant. The figure restricted to the 299 children with coverage information from all three sources, excluding children with DBS collection within 28 days of vaccination. Lines indicate 95% confidence intervals. Estimates are survey weighted.

Of surveyed one-year-old children, 90% in Mexico and 83% in Nicaragua had health cards available at the time of interview. In Mexico, 54%, 86%, and 69% of participants provided information on measles immunization status from recall, health card and DBS, respectively, and 38% reported on all three sources (Fig 1). In Nicaragua, these values were 85%, 80%, and 82%, and 61%. Estimates of measles immunization coverage from interviews (card-based coverage, recall-based coverage and survey-based coverage) ranged from 73% (95% confidence interval [CI]: 68–78%) to 83% (95% CI: 80–87%) for Mexico and from 81% (95% CI: 75–88%) to 85% (95% CI: 79–91%) for Nicaragua (Figs 2 and 3). Effective coverage was statistically significantly lower than survey-based coverage, at 68% (95% CI: 63–73%) and 50% (95% CI: 40–61%) in Mexico and Nicaragua, respectively.

Coverage classification according to health card versus DBS was in agreement for only 77% (95% CI: 74–79%) of children in Mexico and only 61% (95% CI: 55–66%) of children in Nicaragua. Concordance, sensitivity, specificity, and positive and negative predictive values showed poor agreement (Table 3). Of particular concern, 19% (Mexico) and 43% (Nicaragua) of card-positive children did not have antibodies.

**Table 3:** Relationship between crude and effective coverage of measles immunization.

**Mexico**			
**Crude coverage^1^**	**DBS effective coverage**		
	***Yes***	***No***	***Total***
***Yes***	59.8%	14.2%	74.0%
***No***	9.3%	16.7%	26.0%
***Total***	69.1%	30.9%	100%
***Parameters***			
Sample size	895		
Kappa	0.43 (0.39–0.46)		
% agreement	76.5 (73.7–79.3)		
% sensitivity	86.6 (84.4–88.8)		
% specificity	54.0 (50.8–57.3)		
Positive predictive value	80.8 (78.2–83.4)		
Negative predictive value	64.3 (61.2–67.5)		
**Nicaragua**			
**Crude coverage^1^**	**DBS effective coverage**		
	***Yes***	***No***	***Total***
***Yes***	46.7%	35.4%	82.2%
***No***	3.7%	14.2%	17.8%
***Total***	50.7%	49.6%	100%
***Parameters***			
Sample size	299		
Kappa	0.21 (0.10–0.33)		
% agreement	60.9 (55.4–66.4)		
% sensitivity	92.8 (88.8–96.7)		
% specificity	28.6 (20.8–36.3)		
Positive predictive value	56.9 (50.8–63.0)		
Negative predictive value	79.5 (68.0–91.1)		

Table restricted to children with both health card and DBS sources; excluding children with DBS collection within 28 days of vaccination. Estimates are survey weighted.

^1^ Crude coverage: according to child health card.

**Fig 4:**
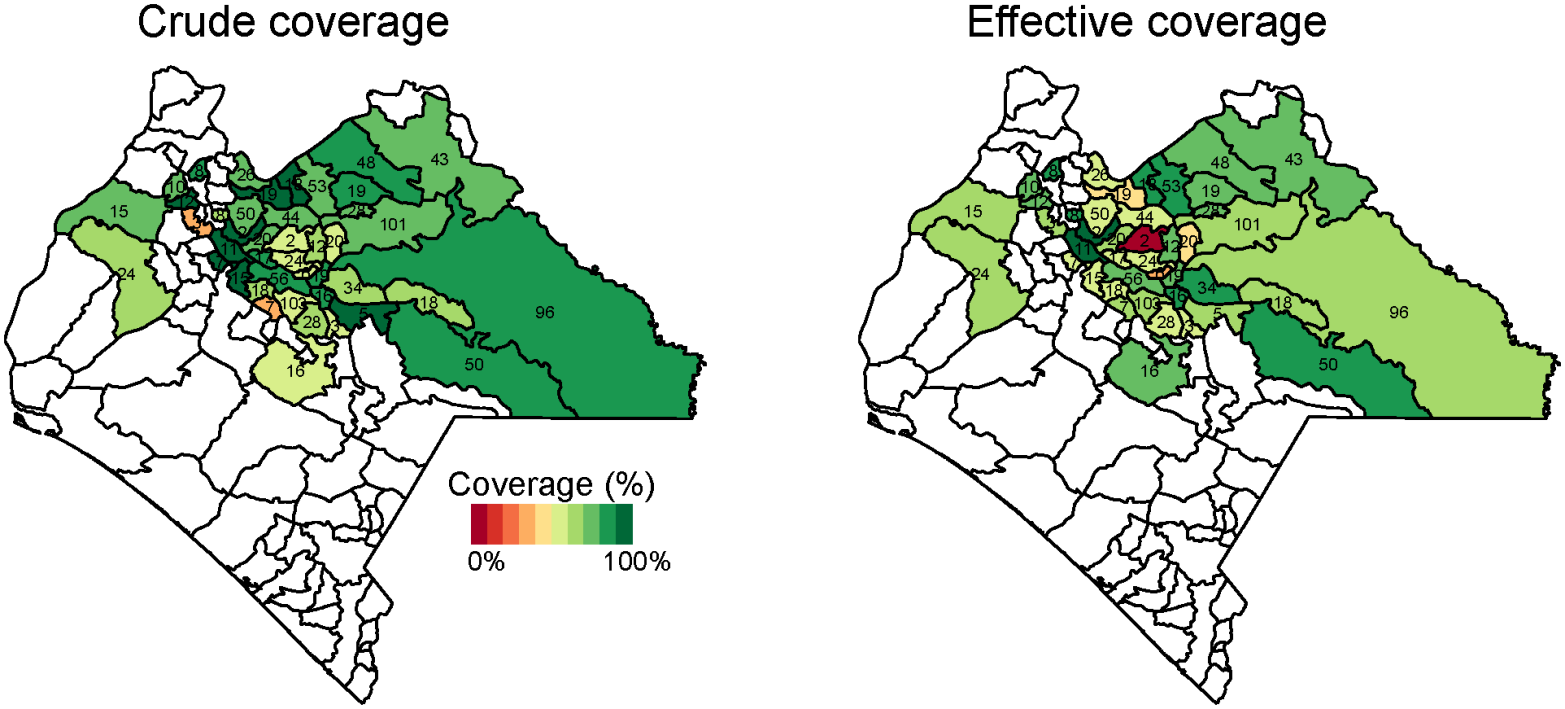
Crude and effective coverage of measles immunization by municipality in Mexico. This figure displays the crude (left) and effective (right) coverage of measles immunization across municipalities in Mexico. Crude coverage tends to be higher than effective coverage, but geographic patterns in high-performing and low-performing areas are similar. The number in each municipality indicates sample size. Estimates are survey weighted.

**Fig 5:**
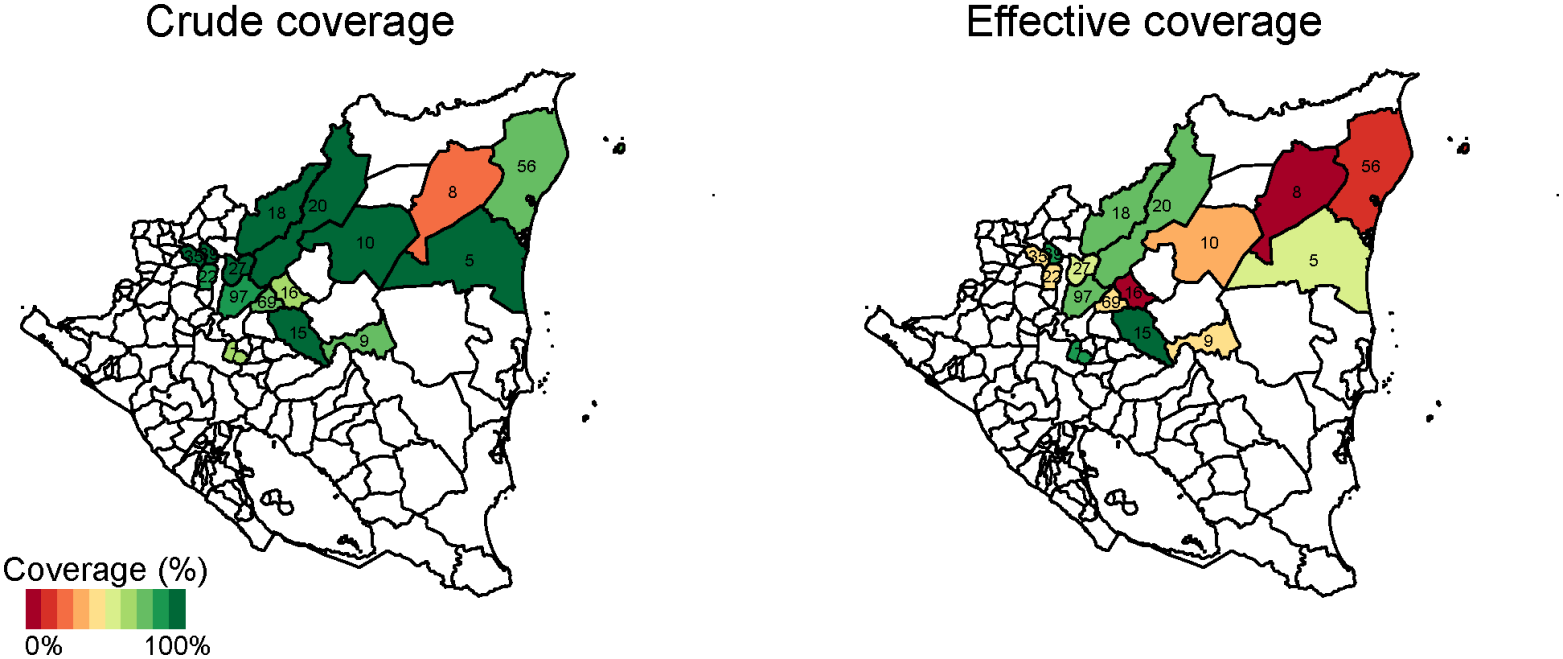
Crude and effective coverage of measles immunization by municipality in Nicaragua. This figure displays the crude (left) and effective (right) coverage of measles immunization across municipalities in Nicaragua. Crude coverage tends to be higher than effective coverage, but geographic patterns in high-performing and low-performing areas are similar. The number in each municipality indicates sample size. Estimates are survey weighted.

In Mexico, crude coverage was highest in the health jurisdiction (administrative service area for the Health Institute of the State of Chiapas) of Meseta Comiteca Tojolabal (89%) and lowest in Valles Zoque (69%) (Figs 4 and 5). Effective coverage was highest in Meseta Comiteca Tojolabal (85%) and lowest in De Los Llanos (53%). In Nicaragua, crude coverage was highest (100%) in Siuna and Prinzapolka, and lowest in Rosita (20%) and Rancho Grande (61%). Effective coverage was highest in Matiguás (92%) and Terrabona (83%), and lowest in Rosita (0%) and Rancho Grande (7%). In general, effective coverage followed geographic patterns similar to those of crude coverage, but at lower levels.

**Fig 6:**
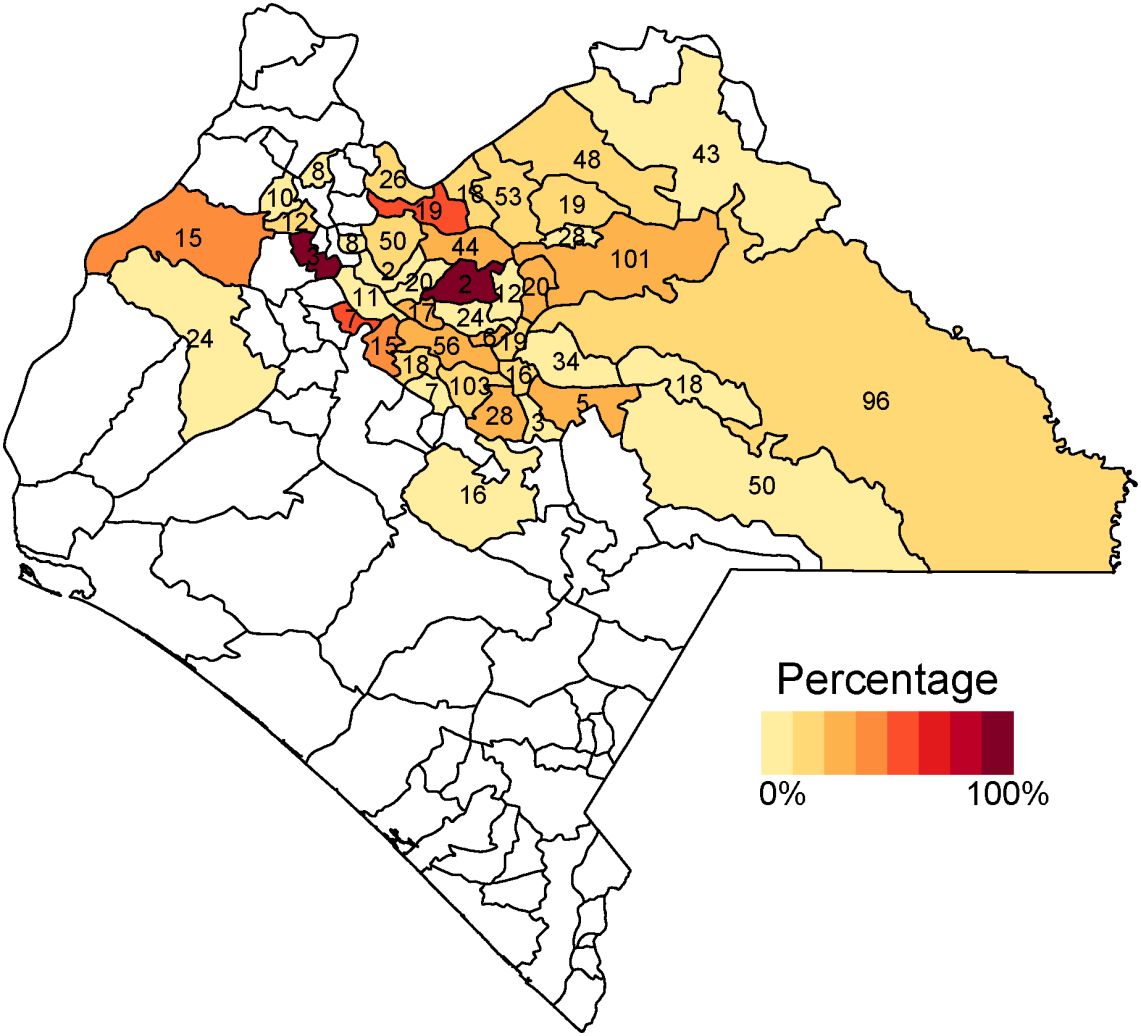
Proportion of card-positive children lacking antibodies in Mexico. This figure displays the proportion of children who lack measles antibodies across municipalities in Mexico, among children with health card documentation of receiving measles vaccination. In both countries, this proportion is very high in small number of municipalities and low in the remaining municipalities. The number in each municipality indicates sample size. Estimates are survey weighted.

**Fig 7:**
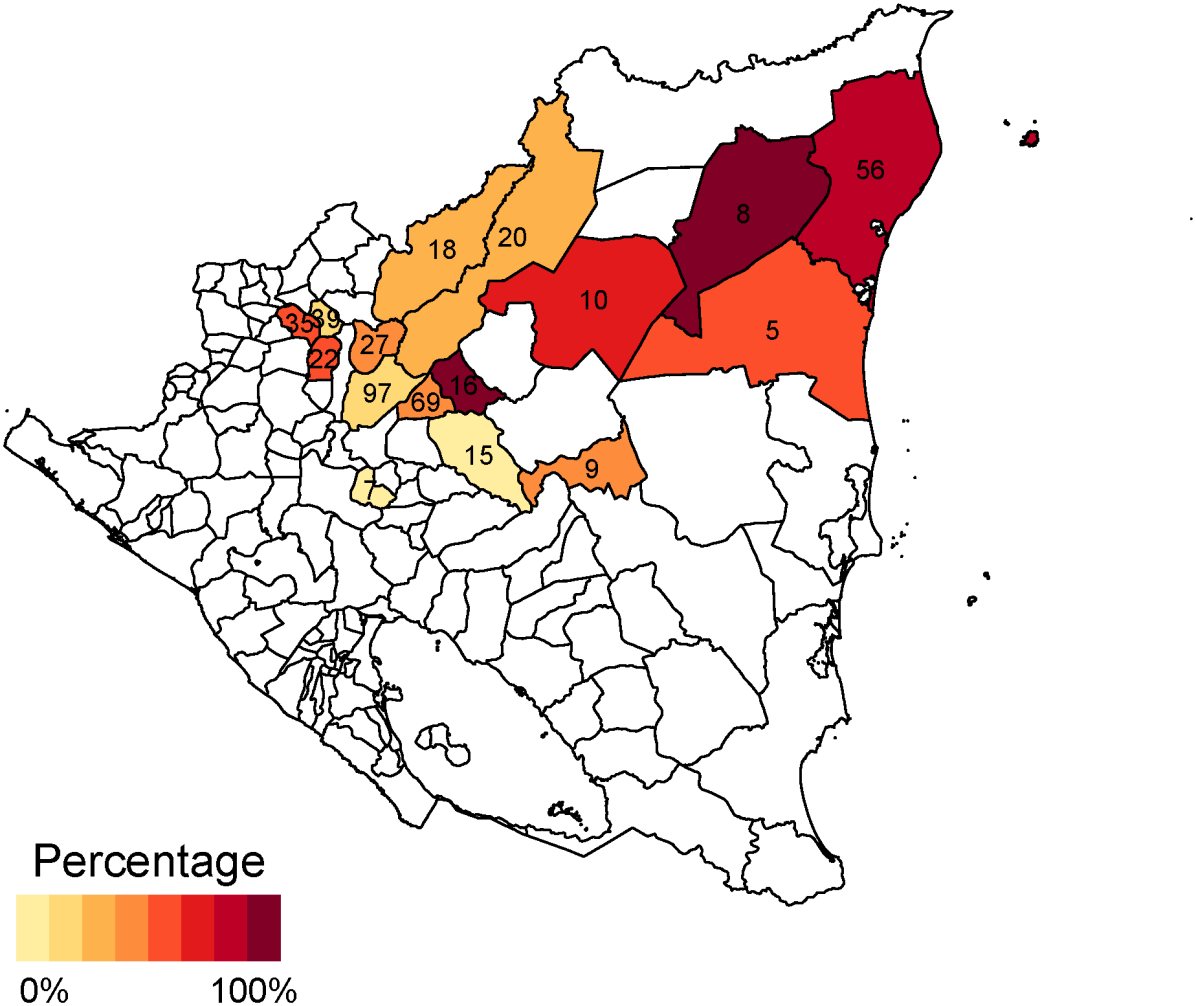
Proportion of card-positive children lacking antibodies in Nicaragua. This figure displays the proportion of children who lack measles antibodies across municipalities in Nicaragua, among children with health card documentation of receiving measles vaccination. In both countries, this proportion is very high in small number of municipalities and low in the remaining municipalities. The number in each municipality indicates sample size. Estimates are survey weighted.

Across municipalities, the proportion of card-positive children lacking antibodies ranged between 0% and 100% in both countries (Figs 6 and 7). Furthermore, there was substantial clustering of these discrepancies; half of card-positive-seronegative cases were concentrated in eight of the 47 surveyed municipalities in Mexico (Chamula, Chilón, Ocosingo, Simojobel, Tila, San Cristóbal de las Casas, Salto de Agua, and Huitiupán), and four of the 16 surveyed municipalities in Nicaragua (Jinotega, Tuma-La Dalia, San Juan Río Coco, and Puerto Cabezas).

In Mexico, few measured individual, maternal, household, or health facility factors explained discrepancies between crude and effective coverage. In multivariate analyses controlling for household and facility characteristics, card-positive children were more likely to lack antibodies if they resided in urban areas or in the jurisdiction of De Los Llanos (Table 4). In sensitivity analyses, findings were not substantively different, except in regression analysis restricted to the subsample of children matching to health facilities by name, in which no covariates were statistically significant.

**Table 4:** Crude and adjusted odds ratios of likelihood that card-positive children lack antibodies in Mexico and Nicaragua.

**Mexico**					
**Characteristic**		**Crude OR**	**p**	**Adjusted OR**	**p**
**Child**	Age < 18 months	0.96 (0.61–1.49)	0.8426	0.89 (0.55–1.43)	0.6354
	Anemic	1.37 (0.80–2.35)	0.2507	1.30 (0.76–2.22)	0.3330
	Underweight	1.02 (0.49–2.12)	0.9681	1.04 (0.49–2.21)	0.9216
**Maternal**	Age (years): 15–19 (ref)				
	20–34	1.90 (0.95–3.79)	0.0709	1.93 (0.89–4.18)	0.1003
	35–49	1.31 (0.75–2.29)	0.3431	1.15 (0.65–2.06)	0.6278
	Literate	0.67 (0.41–1.12)	0.1300	0.62 (0.36–1.04)	0.0719
**Household and community**	Asset score (mean)	0.22 (0.01–4.28)	0.3214	0.44 (0.02–7.90)	0.5812
	Urban	2.23 (1.29–3.85)	0.0047	2.59 (1.36–4.94)	* 0.0047
	Jur: Tulijá Tseltal Chol (ref)				
	Valles Zoque	0.71 (0.10–4.95)	0.7295	0.75 (0.11–5.29)	0.7752
	Mezcalapa	1.30 (0.37–4.64)	0.6836	1.14 (0.28–4.58)	0.8532
	De Los Llanos	0.00 (0.00–0.00)	* <0.0001	0.00 (0.00–0.00)	* <0.0001
	Altos Tsotsil Tseltal	0.70 (0.36–1.36)	0.2918	0.59 (0.28–1.25)	0.1736
	De Los Bosques	1.25 (0.54–2.87)	0.6007	0.92 (0.38–2.23)	0.8577
	Norte	0.48 (0.06–3.97)	0.4936	0.42 (0.06–2.88)	0.3751
	Selva Lacandona	0.70 (0.20–2.41)	0.5723	0.63 (0.16–2.48)	0.5095
	Maya	0.26 (0.07–0.91)	0.0375	0.28 (0.07–1.14)	0.0786
	Meseta Comiteca Tojolabal	0.51 (0.21–1.23)	0.1359	0.52 (0.16–1.64)	0.2657
**Health facility**	# of doctors	1.01 (0.95–1.08)	0.6719	1.03 (0.94–1.12)	0.5763
	Sufficient electrical power	1.05 (0.29–3.81)	0.9468	0.69 (0.13–3.69)	0.6605
	# of refrigerators	1.11 (0.53–2.30)	0.7829	0.94 (0.28–3.20)	0.9265
	1+ broken cold storage observed	1.34 (0.70–2.57)	0.3808	1.36 (0.64–2.91)	0.4222
	Any refrigerator out of temperature range	1.22 (0.49–3.05)	0.6644	1.48 (0.52–4.21)	0.4595
N = 583					
**Nicaragua**					
**Characteristic**		**Crude OR**	**p**	**Adjusted OR**	**p**
**Child**	Age < 18 months	0.70 (0.37–1.32)	0.2745	0.90 (0.45–1.79)	0.7684
	Anemic	1.31 (0.82–2.10)	0.2672	1.36 (0.79–2.34)	0.2661
	Underweight	5.38 (0.54–53.21)	0.1551	7.99 (1.66–38.51)	* 0.0128
**Maternal**	Age (years): 15–19 (ref)				
	20–34	0.62 (0.30–1.29)	0.2048	0.61 (0.26–1.43)	0.2618
	35–49	1.25 (0.62–2.53)	0.5396	2.07 (0.92–4.67)	0.0843
	Literate	0.84 (0.42–1.68)	0.6201	1.53 (0.68–3.46)	0.3090
**Household and community**	Asset score	0.04 (0.00–0.42)	* 0.0097	0.05 (0.00–0.82)	0.0416
	Urban	0.66 (0.23–1.88)	0.4412	0.27 (0.11–0.69)	* 0.0083
	SILAIS: Jinotega (ref)				
	Matagalpa	1.61 (0.63–4.11)	0.3217	0.54 (0.12–2.45)	0.4263
	RAAN	7.83 (2.55–24.03)	*<0.0001	16.59 (2.21–124.53)	* 0.0088
	Madriz	1.27 (0.42–3.83)	0.6764	4.73 (0.90–24.80)	0.0727
**Health facility**	Routine administrative meetings	0.11 (0.01–2.37)	0.1638	0.01 (0.00–1.25)	0.0687
	Doctor on staff	1.56 (0.30–8.02)	0.5950	0.08 (0.00–6.29)	0.2673
	Sufficient electrical power	2.02 (0.52–7.79)	0.3136	3.11 (0.34–28.7)	0.3225
	# of refrigerators	1.27 (0.77–2.07)	0.3519	3.19 (1.07–9.48)	* 0.0427
	1+ broken cold storage observed	0.21 (0.05–0.95)	* 0.0473	7.82 (0.68–90.14)	0.1060
	Any refrigerator not 2–8°C	2.85 (0.19–41.76)	0.4473	0.16 (0.01–3.29)	0.2418
N = 237							

Ref: reference category. Jur: Health jurisdiction. Survey weighted logistic regression. OR: odds ratio. 95% confidence intervals are in parentheses.

* p<0.05.

Covariate definitions: Child age: dichotomous, younger than 18 months or not; anemia: altitude-adjusted hemoglobin of <11.0g/dL; underweight: weight below two standard deviations of World Health Organization 2006 reference weight-for-age; maternal age: 3 age groups: 15–19, 20–34, or 35–49; maternal literacy: full ability to read a written sentence in Spanish; household asset score: proportion of assets inquired about that were owned by the household; urban: surveyed community has population > 2500; health jurisdiction or Local Integrated Healthcare System geographic regions (SILAIS): local health system administrative unit; number of doctors: number of doctors at the health facility attended for immunization; doctor on staff: whether the health facility attended for immunization had a doctor on staff; routine administrative meetings: whether the health facility attended for immunization has routine administrative meetings; sufficient electrical power: whether the health facility attended for immunization has sufficient electrical power to run all of its equipment at all hours; number of refrigerators: number of functional refrigerators owned by the health facility attended for immunization; 1+ broken cold storage observed: whether the health facility attended for immunization has one or more broken cold storage units (refrigerators or cold boxes); and any refrigerator not 2–8°C: whether any refrigerator storing vaccines was out of the appropriate temperature range on the day of the survey (2–8°C).

In adjusted analyses for Nicaragua, card-positive children were more likely to lack antibodies if they resided in rural areas or the North Atlantic region, had low weight-for-age, or attended health facilities with a greater number of refrigerators No other measured individual, maternal, household, or health facility factors were statistically significant predictors. These findings were similar in all sensitivity analyses.

## Discussion

To our knowledge this is the first large study in Mexico or Nicaragua to compare survey-based estimates of measles immunization coverage with the seroprevalence of measles antibodies. Our findings revealed that health card-based estimates of measles immunization coverage are inadequate predictors of immunity. We identified low seroprevalence of protective antibodies against measles in several areas of Mexico and Nicaragua, a finding which calls for immediate action to improve effective coverage. Moreover, crude coverage of full immunization was only 51% in Mexico and 70% in Nicaragua; if the same patterns observed for measles are present for other immunizations, full effective coverage is likely to be extremely low. These findings will enable health authorities to examine and address gaps in crude and effective coverage.

There are several possible reasons why card-positive children might lack antibodies. First, measles vaccine efficacy is imperfect, even under ideal conditions. Previous trials of measles immunization among 12-month-old children in Latin America observed efficacy between 74% and 100% but most were above 93% [31,32]. Underweight (weight for age below minus two standard deviations from median of World Health Organization [WHO] reference population) and recent illness were common among study participants, and whether malnutrition reduces seroconversion rates after measles vaccination is still a source of debate. While a recent WHO report on the immunological basis for measles vaccination concluded that malnourished children have equivalent seroconversion rates [31], at least two studies have identified significant differences in seroconversion depending on nutritional status [32,33]. In fact, in our analyses for Nicaragua, being underweight increased the likelihood that a card-positive child would lack antibodies.

Second, vaccines may be marked on health cards when they were not administered. Benefits from the conditional cash transfer program Oportunidades in Mexico are provided only if children are fully immunized, which might motivate providers to mark vaccines on health cards when not delivered. However, current household enrollment in Oportunidades was not statistically significant in our models. The fact that card-positive seronegative discrepant cases were more prevalent than card-negative seropositive cases suggests that documentation errors were not at random, and therefore some intentional modification of health cards may be present.

Third, interruptions in the cold chain during vaccine transport, storage, or delivery may have rendered vaccines ineffective.,In many of the regions we surveyed transportation and electricity were not dependable. Fourth, there may be limitations in sensitivity of the ELISA or DBS elution methodology employed to test for measles-specific IgG. However, a previous validation of the methods employed in this study identified a test sensitivity of 100% [22]. Fifth, it is possible that some vaccines were prepared or administered incorrectly, resulting in reduced rates of seroconversion. Unfortunately, we could not evaluate this factor using our survey data.

We identified strong geographic patterns in crude and effective coverage. These patterns highlight that some surveyed areas are very high-performing while others experience substantial challenges. This finding also strengthen the validity of the findings—if there were errors in data collection or in the DBS ELISA procedure, we might observe a gap across all municipalities. Instead, we uncovered variable degrees of discordance between coverage measures. We also detected variable levels of coverage, but consistent geographic patterning across sources (i.e. municipalities with low crude coverage tended to have low effective coverage and likewise for high coverage).

Our observation of both very high and very low coverage levels within the same states and demographic groups suggests that health system-level factors rather than individual factors drive the gap between crude and effective coverage. Although it is possible that errors in health card documentation, malnutrition, and low levels of vaccine efficacy may play a role, it is unlikely that these factors account for the entire gap between crude and effective coverage. We suspect that breaks in the cold chain are the main driver of this gap, but limited statistical power inhibited our ability to confirm these findings. Future investigation using spatial analysis methods may achieve better resolution in uncovering these relationships.

We found that card-positive children from urban areas in Mexico were more likely to lack antibodies than children in rural areas. This contrasts with our expectation. One possible explanation is that recent investments in the health insurance program Seguro Popular have strengthened immunization services for the poorest populations in rural but not urban areas. Another explanation is that rural facilities with weak electrical systems are either accustomed to handling electricity shortages or do not store vaccines. Indeed, our interviewers observed electricity challenges in both urban and rural areas, but only 49% of the facilities we surveyed routinely store vaccines, and the majority of these are in urban areas. It is also possible that poor families have moved to urban areas seeking employment and have overwhelmed the health facilities there, causing poor performance. Further investigation is needed to identify the drivers of these patterns.

It is unclear, based on our data, why no measles outbreaks have been observed in either Chiapas, Mexico or in Nicaragua given the low estimated prevalence of protective antibodies (68% and 50% respectively). One possible explanation is that the measles virus has not been recently introduced. Another possible explanation is that, given the rural setting of this study, the observed levels of effective coverage are sufficient to achieve herd immunity [32]. During data collection, field teams did not observe or hear of any measles outbreaks, and when we asked caregivers if their children had ever been diagnosed with measles, only 18 of 1826 said “yes.” After the 1989 measles outbreak, Mexico established an active, sensitive and specific surveillance system which includes a national laboratory network and investigation of all cases of febrile rash illness from weekly health facility reports [2]. This system clearly established that endemic transmission was interrupted in 1996 and is generally regarded as high-quality [2]. Nicaragua’s measles surveillance system has been similarly strengthened with the support of the Pan American Health Organization’s measles eradication initiatives [34]. Still, it is possible that some measles cases may have occurred, but were not captured by surveillance systems. While there were no confirmed cases of measles in Mexico or Nicaragua in 2012 or 2013, between January 1, 2014 and May 5, 2014 there were 1,021 suspected and two confirmed cases in Mexico and 41 suspected cases in Nicaragua [25,26]. The Mexico 2012 National Health and Nutrition Survey also showed recent declines in vaccination coverage [7], which might explain this recurrence. If measles is present, some children may have antibodies from natural exposure rather than immunization, implying that we may have overestimated effective coverage.

### Strengths and limitations

Our study has several limitations. First, we were unable to obtain measles immunization metrics from all three sources (health cards, recall and DBS) for all surveyed one-year old children. For this reason, our findings cannot be generalized to the entire SM2015 population. We also had had limited statistical power to detect significant patterns across demographic, geographic, and health facility factors. However, our findings can be generalized to the population of SM2015 children who would have offered health cards and DBS had they been surveyed. Among this population, our finding that 19% of card-positive children in Mexico and 43% of card-positive children in Nicaragua lacked antibodies is worrisome and notable. It implies that a substantial proportion of children believed to be protected against measles virus are, in reality, at risk. Our finding of significant gaps between card-based and serology-based measures of protection among surveyed children alone merits immediate response and further investigation.

Second, our ability to match participants to health facilities was limited. Due to the geographic score and focus of the SM2015 evaluation, we did not survey all health facilities serving the study population and hence could only like 25–30% of children to surveyed health facilities. While we imputed missing values using the average characteristics of surveyed facilities serving the area of residence of the child, health facility data should be interpreted with caution, and future research should examine alternative methods to coordinate such data. Finally, our data are based on self-reported information from a household survey and may be subject to self-reporting bias.

This study also has several important strengths. First, we captured data from a large, representative sample of children in poor areas. In contrast with the most recent health and nutrition surveys in Mexico [6] and Nicaragua [10] which collected immunization data from 723 and 223 children aged 12–23 months in SM2015 areas, we captured 1,134 and 454, respectively, allowing for greater statistical precision. Second, we used electronic data capture and standardized methodology during data collection. These processes enhanced data quality by allowing us to monitor surveys and provide feedback to field teams in real time. Third, we conduct our own census in selected primary sampling units. In contrast with many national surveys which use the most recent national census as the sampling frame which can be outdated, we established the most accurate and up-to-date sampling frame to assure that we captured everyone in the target population. Fourth, we employed a DBS ELISA test procedure that had been previously validated in the same laboratory with the same reagents and technicians [22]. The process assured that assay results were as accurate and sensitive as possible compared to the validation.

### Conclusion

Our findings are crucial to immunization activities in Mexico and Nicaragua. Our results show that the exclusive use of recall and card-based measures of coverage is unwise. While no best method for evaluating immunization program performance is established, our study calls for the use of seroprevalence as a primary metric for evaluating vaccination programs. Such an approach will help ensure the health and wellbeing of children in Mesoamerica. To achieve and maintain high levels of protection against measles and other vaccine-preventable diseases, it is necessary to identify gaps in effective coverage, not just crude coverage. We raise several possible explanations for gaps in effective coverage and encourage monitoring of the vaccine cold chain and inspection of vaccine integrity. These steps will help ensure that high-quality immunizations are used to achieve high effective coverage of measles immunization in Mexico. At a time when public health systems are seeking to eliminate certain vaccine-preventable diseases, we should apply effective not crude measures to monitor and evaluate our efforts.
